# Loss of protein phosphatase 2A regulatory subunit *PPP2R5A* is associated with increased incidence of stress-induced proarrhythmia

**DOI:** 10.3389/fcvm.2024.1419597

**Published:** 2024-05-28

**Authors:** Florentina Pluteanu, Dennis Glaser, Fabian Massing, Jan S. Schulte, Uwe Kirchhefer

**Affiliations:** ^1^ Department of Anatomy, Animal Physiology and Biophysics, University of Bucharest, Bucharest, Romania; ^2^ Institut für Pharmakologie und Toxikologie, Universitätsklinikum Münster, Universität Münster, Münster, Germany

**Keywords:** protein phosphatase 2A, *PPP2R5A*/B56α, electrocardiogram, monophasic action potential, Ca^2+^ transients, ventricular tachyarrhythmia, K^+^ channel currents

## Abstract

**Background:**

Protein phosphatase 2A (PP2A) is a serine/threonine-selective holoenzyme that controls Ca^2+^ homeostasis and contractility of the heart via dephosphorylation of regulatory proteins. In some genetically modified mouse models with increased arrhythmogenicity, a reduced expression of the regulatory subunit B56α of PP2A was found as a concomitant effect. Whether there is a general correlation between the abundance of B56α and the promotion of cardiac arrhythmogenesis remains unclear.

**Methods:**

The aim of this study was therefore to investigate the role of PP2A-B56α in the propensity for arrhythmic activity in the heart. The experimental analysis of this question has been addressed by using a mouse model with deletion of the PP2A-B56α gene, *PPP2R5A* (KO), in comparison to wild-type animals (WT). Evidence for arrhythmogenicity was investigated in whole animal, isolated heart and cardiomyocytes by ECG, recording of monophasic action potential (MAP) induced by programmed electrical stimulation (PES), measurement of Ca^2+^ transients under increased pacing frequencies and determination of total K^+^ channel currents (*I*_K_).

**Results:**

ECG measurements showed a prolongation of QT time in KO vs. WT. KO mice exhibited a higher rate of premature ventricular contractions in the ECG. MAP measurements in Langendorff-perfused KO hearts showed increased episodes of ventricular tachyarrhythmia induced by PES. However, the KO hearts showed values for MAP duration that were similar to those in WT hearts. In contrast, KO showed more myocardial cells with spontaneous arrhythmogenic Ca^2+^ transient events compared to WT. The whole-cell patch-clamp technique applied to ventricular cardiomyocytes revealed comparable peak potassium channel current densities between KO and WT.

**Conclusion:**

These findings support the assumption that a decrease or even the loss of PP2A-B56α leads to an increased propensity of triggered arrhythmias. This could be based on the increased spontaneous Ca^2+^ tansients observed.

## Introduction

Ventricular (tachy)arrhythmia (VT) is a common cardiac arrhythmia associated with heart failure and is associated with a high mortality rate if left untreated (1). In addition to heart failure, myocardial ischemia, various drugs or congenital ion channel mutations can also lead to VTs. A distinction is made between monomorphic and polymorphic VTs according to the shape of the ventricular complexes, each of which is based on different electrophysiological causes. The increased tendency of ventricular cardiomyocytes to generate delayed afterdepolarizations (DADs) is currently regarded as an important cause of VTs (1). These in turn are attributed to the occurrence of spontaneous extrasystolic Ca^2+^ release from the sarcoplasmic reticulum (SR) (2, 3). The triggers for an increased Ca^2+^ release from the SR may be an increased influx of Ca^2+^ ions via the LTCC, an increased Ca^2+^ content of the SR or an increased open probability of the cardiac ryanodine receptor (RyR2) (4). The activity of RyR2 is primarily determined by its degree of phosphorylation (5, 6) It has been shown that greater phosphorylation of the Ca^2+^ release channel is associated with an increased propensity for triggered arrhythmias and VTs (7). β-adrenergic stimulation can increase the extent of RyR2 hyperphosphorylation and increased open probability, as observed in catecholaminergic polymorphic ventricular tachycardia (8).

Protein phosphatases (PP) in turn ensure the balance of the degree of phosphorylation at the RyR2. Research has focused on the regulation of intracellular Ca^2+^ handling in healthy and diseased myocardium by PP1 in recent decades (9). It has been shown that PP1 is involved in the regulation of almost every step of the cardiac Ca^2+^ cycling machinery. Of particular importance is the spatio-temporal control of the Ca^2+^ regulatory proteins of the SR. A disruption of this control through altered expression or function of PP1, which in turn usually results in reduced phosphorylation levels of target proteins, is considered to be responsible for the development of heart failure or atrial fibrillation (9, 10). PP1 is part of a macromolecular RyR2 complex that also contains FK506-binding protein 12.6, protein kinase A (PKA), the PKA regulator subunit RII, and muscle A-kinase anchoring protein (mAKAP). This complex reduces the SR Ca^2+^ leak in response to β-adrenergic stimulation via dephosphorylation of specific phosphorylation sites on RyR2 (11). However, although less studied so far, PP2A is also part of the SR Ca^2+^ release apparatus. It reverses the process of phosphorylation and ensures reduced RyR2 activity (12). The target or substrate activity of PP2A is in turn dependent on the association of the core enzyme of structural A and catalytic C subunit with a unique regulatory B subunit (13, 14). It is currently known that PP2A is localized to the RyR2 complex via B56α*/PPP2R5A* (B' gene family) (12). In addition to RyR2, it has also been shown that B56α-associated PP2A can decrease the PKA-dependent phosphorylation level of the LTCC, resulting in lower *I*_CaL_ and reduced inward (depolarizing) current (15). It is clear from this that reduced expression and/or activity of PP2A-B56α could lead to potentially dangerous ventricular tachyarrhythmias, particularly following catecholaminergic stress via RyR2 hyperphosphorylation. Two studies to date have indirectly demonstrated that an increase in arrhythmogenicity in mouse models is associated with the dissociation of B56α-coupled PP2A activity at RyR2 (16, 17).

To test whether a direct decrease in PP2A-B56α expression/activity translates into an increased propensity for VTs, we utilized our *PPP2R5A*/B56α deletion model (18). This model shows a decrease in PP2A, hyperphosphorylation of RyR2 after administration of isoprenaline and corresponding changes in SR Ca^2+^ cycling, all potential prerequisites for an increased predisposition to VTs. To clarify the question, ECGs, MAP measurements in the isolated heart after PES as well as in single heart muscle cells under β-adrenergic stimulation and finally Ca^2+^ transients in isolated cells under different stimulation frequencies were performed in the mouse model.

## Materials and methods

### Animal model

Knockout mice with global deletion of the *PPP2R5A* gene were generated as previously described (18). All experiments were performed on 20- to 24-week-old mice. Balanced proportions of both sexes were always used. KO and WT mice were bred on a C57BL/6 strain background. All animal handling and maintenance procedures were conducted in accordance with approved protocols by the animal welfare committee of the University of Munster and the LANUV (*N*RW, Germany; ID#84-02.04.2014.A485). These protocols also complied with the National Institutes of Health Guidelines for the Care and Use of Laboratory Animals.

### ECG measurements

**Figure 1:**
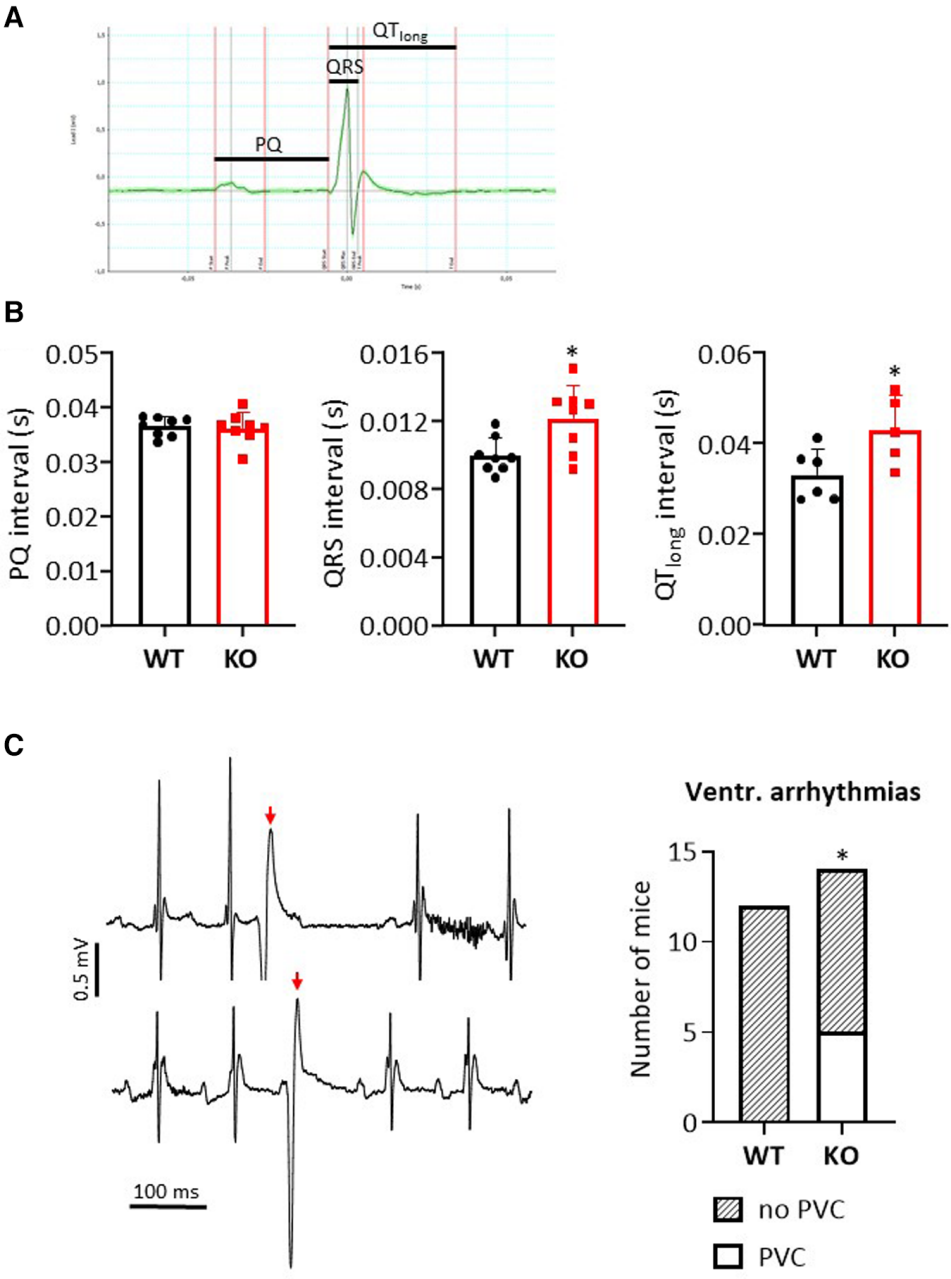
Loss of B56α results in premature ventricular contractions in ECG measurements. (**A**) Representative representation of an ECG recording in a WT mouse. The indicated bars mark the analyzed intervals. (**B**) Data are shown for the PQ, QRS and QTlong interval in comparison of KO to WT mice (*P < 0.05 vs. WT, *N* = 8 mice each, unpaired Student's *t*-test). (**C**) Exemplary representation of a section of two independent ECG measurements in KO mice (left panel) with marking of premature ventricular contractions (PVCs, red arrow). The summed data show an occurrence of PVCs only in the KO mice (right panel) (*P < 0.05 vs. WT, Fisher's exact test).

The measurement was carried out according to an established protocol (19). First, the mice were anaesthetized with 1.5% isoflurane. After a pain response test, the ECG electrodes were placed subcutaneously in the Einthoven positions on the extremities of the animals (20, 21). The measurement was recorded basally for 5 min using a PowerLab/Dual Bio Amp and the LabChart Pro software. The ECG parameters were also evaluated using the LabChart Pro V7 software. The individual parameters and intervals (Figure 1A) were evaluated manually. No standardized definition has yet been established for determining the duration of excitation recovery. In mice, the QRS complex is immediately followed by a positive J-wave, which is then followed by an often negative T-wave, particularly in anesthetized mice (22, 23). Therefore, the so-called QT_long_ time was determined (24). This was defined as the distance from the beginning of the Q-wave to the point where the negative T-wave reaches the isoelectric line again. Due to the comparability of the RR interval under spontaneous heartbeat, the QT time was not normalized using the adapted Bazett formula for mice (25). To potentially induce proarrhythmic events, 4 mg/kg isoprenaline was administered intraperitoneally to the mice. Further observation for such events was carried out for a duration of 15 min per animal.

### Preparation of Langendorff-perfused hearts

For electrophysiological measurements on the isolated organ, Langendorff-perfused mouse hearts were used as previously described (26). Hearts were quickly removed avoiding or minimizing contact with the heart tissue and then placed in an ice-cold Krebs-Henseleit solution containing (mM): 119 NaCl, 25 NaHCO_3_, 4 KCl, 1.2 KH_2_PO4, 1 MgCl_2_, 1.8 CaCl_2_, 10 glucose, and 2 Na-pyrovate (pH = 7.4). The solution was gassed with 95% O2/5% CO2. Under the ice-cold buffer, excess tissue surrounding the heart was removed, leaving a 2–3 mm section of the aorta. The aorta was cannulated (20 Gauge) and looped with a suture. The cannula was filled with ice-cold buffer using a syringe. The heart thus supplied was then transferred to the Langendorff system and retrogradely perfused with the buffer described above. The buffer was kept at 37°C via a water jacket with a pump system. The isolated heart was supplied with a constant flow of approx. 2 ml per min using a peristaltic pump. The cannulated hearts were perfused for 20 min before further testing. Under these conditions, living healthy hearts achieved uniform red coloration and spontaneous rhythmic contractions. Hearts that did not achieve these characteristics were discarded for further testing.

### Monophasic action potential recording

Monophasic action potentials (MAPs) of the ventricles were recorded using an established contact electrode technique (27). Epicardial MAPs were recorded on spontaneously beating hearts from the surface of the left ventricular epicardium using a silver chloride recording electrode (Hugo Sachs Elektronik). Signals obtained from the recording electrode were amplified and low-pass filtered between 0.1 Hz and 1.0 kHz using a standard amplifier and digitized using an analogue to digital converter (Hugo Sachs Elektronik). MAP waveforms were analyzed using OriginPro software (OriginLab Corporation). MAPs used for analysis showed satisfactory established waveform characteristics including a stable baseline, a fast upstroke phase with sustained amplitude, a slightly contoured repolarization, a stable duration, and a complete repolarization reaching baseline (28). The MAP waveforms were used to obtain the action potential durations (APDs) at 70% (APD_70_) and 90% (APD_90_) repolarization.

### Programmed electrical stimulation

Programmed electrical stimulation (PES) was used to assess ventricular arrhythmogenesis in Langendorff-perfused isolated hearts and recorded as MAPs (29). The PES protocol stimulated the hearts using an IonOptix MyoPacer. We started by using cycles each consisting of a 10 stimuli (S1) drive train with a frequency of 15 Hz (S1-S1) followed by an extra-stimulus (S2). The first S1-S2 interval (between the 10th S1 and first S2 stimuli) was set to 20 Hz. Successive cycles progressively reduced the S1-S2 interval by elevating the frequency until the S2 stimulus could no longer evoke a ventricular deflection, at which point, the whole heart preparation reached the ventricular effective refractory period (VERP). VERP was hence defined as the longest S1-S2 interval that could not elicit a ventricular deflection. This procedure was extended accordingly up to the application of a fourth stimulus (S4). The PES-induced episodes of ventricular tachyarrhythmias (VTs) were counted for the pacing intervals S1-S2, S1-S2-S3 and S1-S2-S3-S4 individually and their total number and analyzed for their morphology (monomorphic vs. polymorphic) and duration (sustained vs. non-sustained).

### Isolation of cardiomyocytes

Isolation of ventricular cardiomyocytes followed an approved protocol (30). Mice were killed by cervical dislocation, and the heart was immediately removed. The cannulated heart was then connected to a modified Langendorff apparatus and retrogradely perfused for 5 min with a perfusion buffer (in mM: NaCl 113, KCl 4.7, KH_2_PO_4_ 0.6, Na_2_HPO_4 _×_ _2H_2_O 0.6, MgSO_4 _×_ _7H_2_O 1.2, NaHCO_3_ 12, KHCO_3_ 10, taurine 30, glucose 11.1, BDM 10, HEPES 10, pH = 7.45) at a constant flow rate of 2.5 ml/min. For another 7.25 min, digestion was performed with an enzyme solution containing Liberase DH. The aorta and atria were then separated from the heart. The remaining heart was minced in a 5 ml enzyme stop solution (10% neonatal bovine calf serum, 12.5 µM CaCl_2_ in perfusion buffer). After 10 min of incubation, the sedimented pellet was transferred into 10 ml of the enzyme stop solution (containing only 5% neonatal bovine calf serum), filtered through nylon gauze, and centrifuged at 500 rpm for 1 min. The cell pellet was then resuspended in 10 ml of perfusion buffer and the Ca^2+^ concentration was raised stepwise to 1 mM. Finally, centrifugation was performed again for 1 min at 500 rpm, and the cell pellet was resuspended in perfusion buffer containing 1 mM CaCl_2_. Cardiomyocytes were kept at room temperature and used within 6 h after isolation.

### Single cell action potential measurements

Action potentials (AP) were measured in Ca^2+^ tolerant ventricular myocytes using amphotericin B (240 µg/ml) perforated patch-clamp technique in current clamp mode. All recordings were performed at room temperature (22-24°C, RT). Borosilicate glass capillaries (Science Products, Hofheim, Germany) were pulled to a resistance of 3-5 M*Ω*. Data were acquired using an EPC-800 amplifier and digitized with an 18bit A/D converter InstruTech ITC-18 under the control of the PatchMaster software (HEKA Elektronik, Lambrecht, Germany). The bath solution contained (in mM): 136 NaCl, 5.4 KCl, 1 CaCl_2_, 2 MgCl_2_, 5 HEPES, 0.33 NaH_2_PO_4_ and 10 glucose, pH 7.4, and the pipettes were filled with a solution containing (in mM): 5 NaCl, 120 KCl, 2.5 MgATP, 1 EGTA and 5 HEPES pH 7.2. The AP recordings were initiated at access resistances bellow 30 MΩ. The average access resistance reached for AP recordings was not different between genotypes: 17.3 ± 1.7 MΩ for WT myocytes (*n* = 18) and 18.3 ± 1.5 MΩ for KO myocytes (*n* = 19). To trigger the AP, myocytes were stimulated for 20 s at 1 Hz frequency using supra-threshold stimuli of 3–6 ms duration. Three to five consecutive AP traces at steady state were averaged and AP duration was measured from the peak to 50%, 70% and 90% repolarization. For this study we used ventricular myocytes presenting clear striations, rectangular shape and with no signs of arrhythmia. The average membrane capacitance [Cm, expressed in picofarads (pF)] was not different between genotypes: 217 ± 11 pF (WT, *n* = 18) and 199 ± 13 pF (KO, *n* = 19, NS).

### Potassium current recordings

Whole-cell outward potassium currents (*I*_K,total_) were recorded in ventricular myocytes. The cells were chosen as described above, with an average Cm of 202.8 ± 13 pF (WT, *n* = 13) and 205.2 ± 12 pF (KO, *n* = 17, NS). Measurements were performed using amphotericin B perforated patch clamp technique in voltage clamp mode, as described above. For the voltage clamp recordings, the series resistances (19.4 ± 1.3 MΩ, *n* = 13 WT myocytes, and 18.05 ± 1.3 MΩ, *n* = 17 KO myocytes, NS) were at least 55% compensated. The cells were clamped at −80 mV, followed by a 10 milliseconds prepulse to −40 mV (to inactivate voltage dependent sodium channel) before the application of a series of voltage steps in 10 mV increments from −80 mV to +70 mV for a duration of 1 s. Current amplitudes (expressed in picoamperes, pA) were measured as the peak current at the beginning of the depolarizing pulses minus the mean current at −40 mV. The currents were normalized to Cm (expressed in pF) of the cell to obtain the current density (pA/pF) and ploted vs. the corresponding voltage to obtain current-voltage relations (IV plots). Current voltage (IV) relations obtained from the cells of the same genotype were averaged. The mean ± SD calculated for each genotype were plotted for comparison.

### Ca^2+^ transients

Intracellular Ca^2+^ cycling of cardiomyocytes was studied by use of a dual emission photometry system combined with a CCD camera (Myocyte Calcium Recording System; IonOptix Ltd.) attached to an epifluorescence microscope (Nikon). Cardiomyocytes were initially incubated with 23.3 µM Indo-1/AM (Molecular Probes) for 10 min in a perfusion chamber mounted on the microscope. This chamber was perfused with bath solution composed of (in mM): 140 NaCl, 5.8 KCl, 0.5 KH_2_PO_4_, 0.4 Na_2_HPO_4_, 0.9 MgSO_4_, 10 HEPES, 1 glucose, and 2 CaCl_2_, pH = 7.45. Thereafter, cardiomyocytes were field-stimulated under basal conditions with 0.5 Hz as well as under increasing stimulation frequencies up to 3 Hz. The emitted fluorescence was recorded at wavelengths of 405 and 495 nm. The ratio of both wavelengths was taken as an index of cytosolic Ca^2+^ concentration.

### Data analysis

The results are presented both in terms of individual data points and as means with corresponding standard deviations (±SD), if not indicated otherwise. In the context of single-cell measurements, the data points represent individual cardiomyocytes (“n”), whereas in the context of multicellular measurements, the data points correspond to individual hearts or mice (“N”). Statistical analyses were conducted using either GraphPad Prism (Graphpad Software Inc.) or SigmaPlot software (Systat Software GmbH). Statistical significance was tested against WT controls within the same condition. Normally distributed data were analyzed with an unpaired two-tailed Student *t*-test, while non-normally distributed data were analyzed with a Mann–Whitney *U*-test. The Fisher exact test or *χ*^2^ test were used for the analysis of frequency distributions depending on the sample size. Total outward K^+^ currents were analyzed by a two-way repeated measures ANOVA. A *p*-value <0.05 was considered statistically significant.

## Results

### Occurrence of premature ventricular contractions in KO mice

The effects of B56α deletion on the electrical activity of the heart were determined by ECG for individual parameters (Figure 1A). PQ time was unchanged between genotypes (Figure 1B). In contrast, the QRS interval was increased by 22% in KO compared to WT hearts (Figure 1B). QT time was also prolonged by 30% in KO compared to WT hearts (Figure 1B). The interval from the starting point of the Q-wave to the second intersection of the T-wave with the baseline was used to determine the QT time. Intraperitoneal application of isoprenaline maintained the changes between KO and WT after 90 s as observed under basal conditions (QRS interval: 0.013 ± 0.0018 vs. 0.0104 ± 0.0017 s, resp., *N* = 8 each, *P *< 0.05; QT time: 0.0374 ± 0.0046 vs. 0.0305 ± 0.0017 s, resp., *N* = 8 each, *P *< 0.05). Whether the increase in QRS complex and QT time are related to the moderate increase in heart weights remains unclear (18). However, it has already been shown that a prolonged QT time is associated with an increase in proarrhythmic activity (31). Indeed, we observed premature ventricular contractions under catecholaminergic stress in KO mice, but not in any of the wild-type animals examined (Figure 1C). However, overall the arrhythmogenic phenotype in KO was mild, as only a maximum of 2 PVCs were recorded per affected animal. All findings should be seen against the background of an unchanged heart rate both under basal conditions and after catecholamine administration between the two genotypes (18).

### Increased rate of sustained VTs in isolated Langendorff-perfused KO hearts

**Figure 2:**
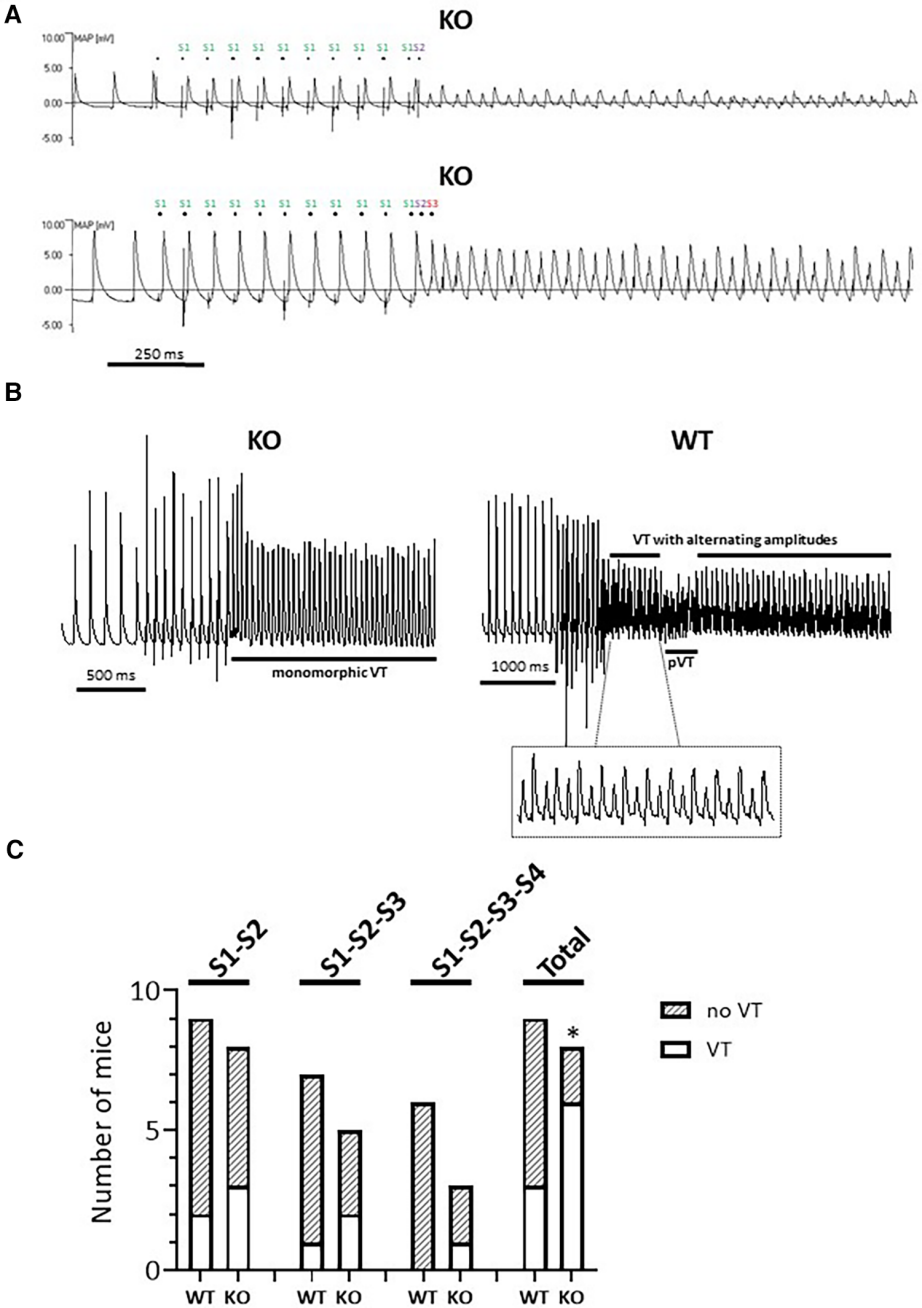
Higher rate of VTs in isolated Langendorff-perfused KO hearts. (**A**) Representative representation of a MAP recording with provoked electrical stimulation (PES) in two independent KO hearts. Both the regular (S1) and the extra provoked stimuli (S2 or S2-S3) are marked. (**B**) Examples of MAP recordings with constant (left panel, monomorphic) and alternating amplitudes (right panel). The VT phases with alternating amplitude (see inset) deflections are interrupted by a polymorphic phase (pVT). (**C**) PES-induced episodes of VTs appeared in the KO hearts under all stimulation conditions, but not in WT with three additional stimuli. Quantification is shown for all PES protocols and in their entirety (total) (*P < 0.05 vs. WT, *χ*^2^ test).

The analysis of MAP recordings allows a closer differentiation of hearts with arrhythmogenic and non-arrhythmogenic phenotypes as well as the observation of a monomorphic vs. polymorphic progression. MAP waveforms were therefore compared between genotypes during PES in a second series of experiments on isolated Langendorff-perfused hearts. Figure 2A shows a typical MAP recording under spontaneous heartbeat and under pacing. In each of the two panels, dots above each MAP recording indicates the S1 stimulus. The traces also show the corresponding stimulus artifacts in the regular cycles, followed by the elicited MAPs. MAPs in response to S1 stimuli were consistent in waveform, with a stable baseline, rapid upstroke, and smooth repolarization (32, 33). Spontaneous events, such as early or late afterdepolarizations, were not observed in either genotype. However, as shown here for two different KO hearts (Figure 2A), episodes of VTs occurred after an S2 (upper panel) or S2-S3 (lower panel) extra-stimulus. Both VTs with alternating (Figure 2A, lower panel; Figure 2B, right panel) and constant (monomorphic) amplitude (Figure 2B, left panel) occurred. We were also able to observe courses with interspersed phases of polymorphic VTs (Figure 2B, right panel). Overall, including all stimulus intervals, there was an increased rate of VTs in KO compared to WT hearts (Figure 2C), with the greatest number of VTs occurring during the S2 extra stimulus. In the WT animals, 2 of 3 hearts showed both VTs with alternating amplitudes and phases of polymorphic VTs. In KO, 4 of 6 hearts showed VTs with alternating amplitudes and two hearts showed polymorphic VTs. In both genotypes, two hearts showed sustained VTs (duration of VTs > 30 s).

### Comparable AP duration between KO and WT

**Figure 3:**
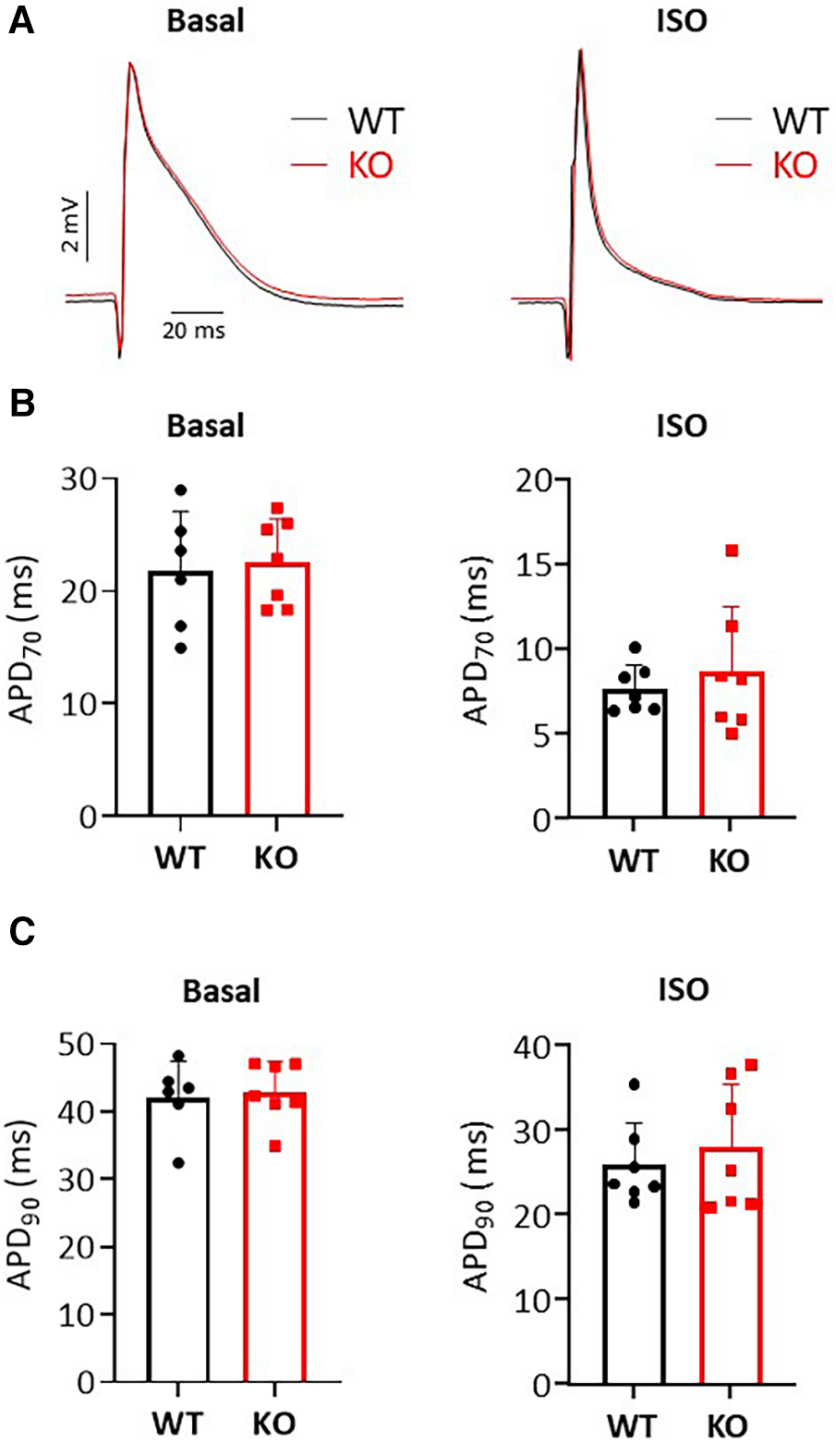
Unchanged MAP durations in isolated Langendorff-perfused hearts. (**A**) Representative representation of MAP recordings under basal and isoprenaline (ISO) stimulated conditions in KO and WT hearts. The application of catecholamines causes a shortening of the MAP duration. (**B,C**) Shown is the duration of MAP at 70% and 90% repolarization under baseline and after ISO administration in both genotypes (*N* = 6–7 WT and 7 KO hearts, n.s.).

**Figure 4:**
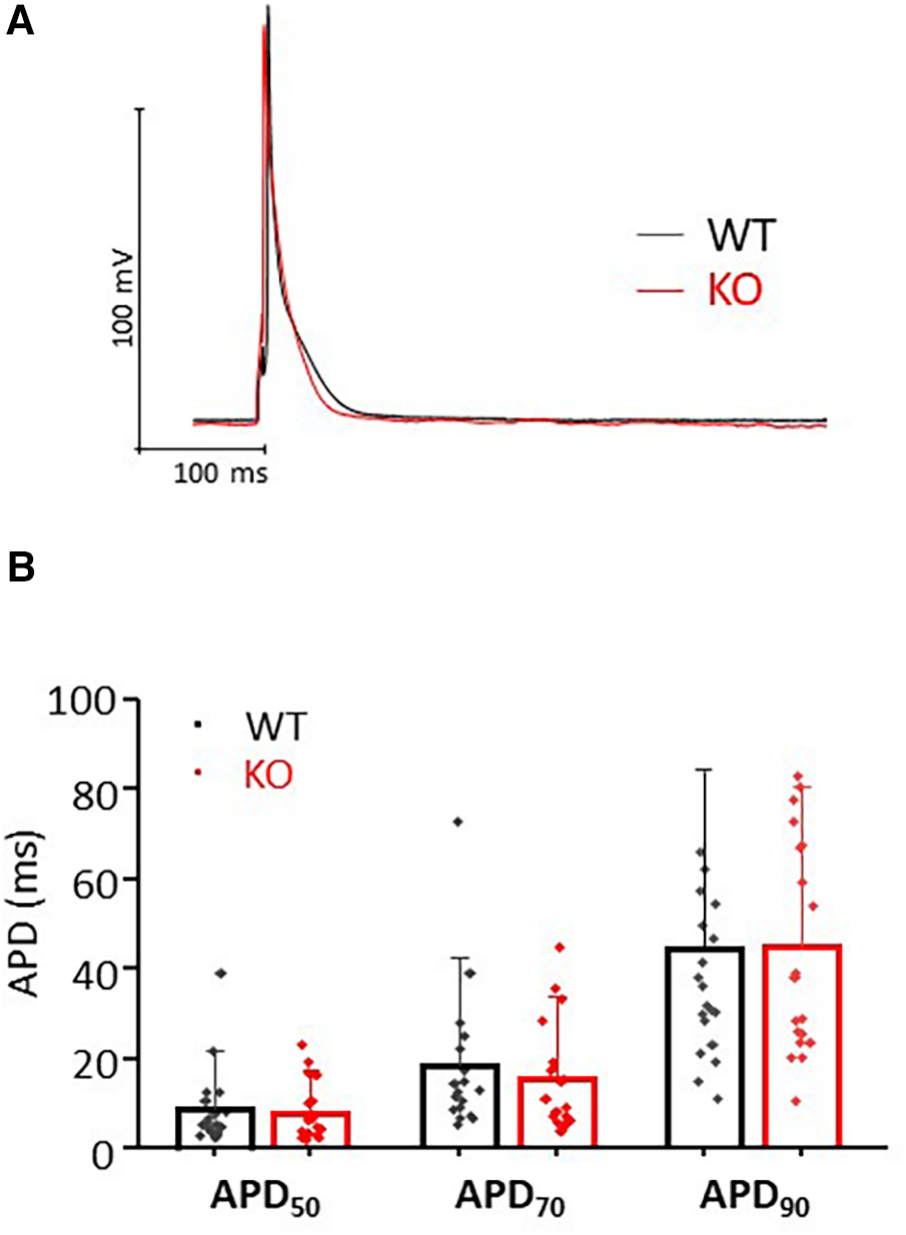
Comparable AP durations in isolated cardiomyocytes. (**A**) Representative representation of AP traces in KO and WT cells. (**B**) AP duration was determined at 50%, 70% and 90% repolarization in both genotypes (*n*/*N* = cardiomyocytes/mice = 18/6 and 19/7 from WT and KO, respectively, n.s.).

**Figure 5:**
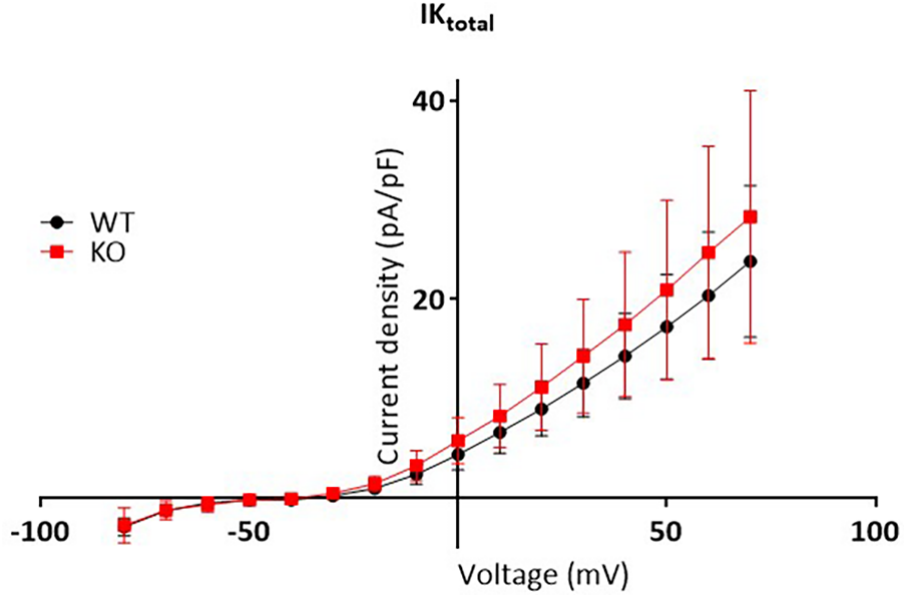
Unchanged total outward K^+^ current between genotypes. Total outward K^+^ curents measured in isolated myocytes as the peak outward current at the beginning of the depolarizing pulse, were normalized to membrane capacitance to calculate the current density (pA/pF) and plotted vs. the corresponding voltage step (mV). The graph shows IV plots. The points represent mean ± SD of 13 WT cells (black symbols) and 17 KO cells (red symbols) isolated from *N* = 7 WT and 6 KO mice, respectively. Two way repeated measures ANOVA with Sidak’ multiple comparisons test estimated no differences in current densities between genotypes.

To test whether changes in repolarization duration, particularly under β-adrenergic stress, are responsible for the observed propensity for VTs in KO, we determined epicardial MAPs in isolated hearts (Figure 3A). However, comparison of epicardial recordings showed that action potential duration (ADP) at 70% was comparable between KO hearts and WT both under basal conditions and after application of isoprenaline (Figure 3B). The repolarization values at 90% APD duration also showed no differences between the genotypes (Figure 3C). In addition, AP duration was also determined in isolated cardiomyocytes (Figure 4A). For all parameters determined, no differences were measured between KO and WT cardiomyocytes under basal conditions (Figure 4B). Whether the difference between prolonged ECG parameters and an unchanged MAP duration relates to a possible dispersion of the spread of excitation to different regions of the ventricular myocardium (34) remains to be elucidated. However, in agreement with the unchanged repolarization time, the peak outward K^+^ current (*I*_K,total_) was also comparable between the genotypes (Figure 5).

### Arrhythmic Ca^2+^ transient events in KO cardiomyocytes

**Figure 6:**
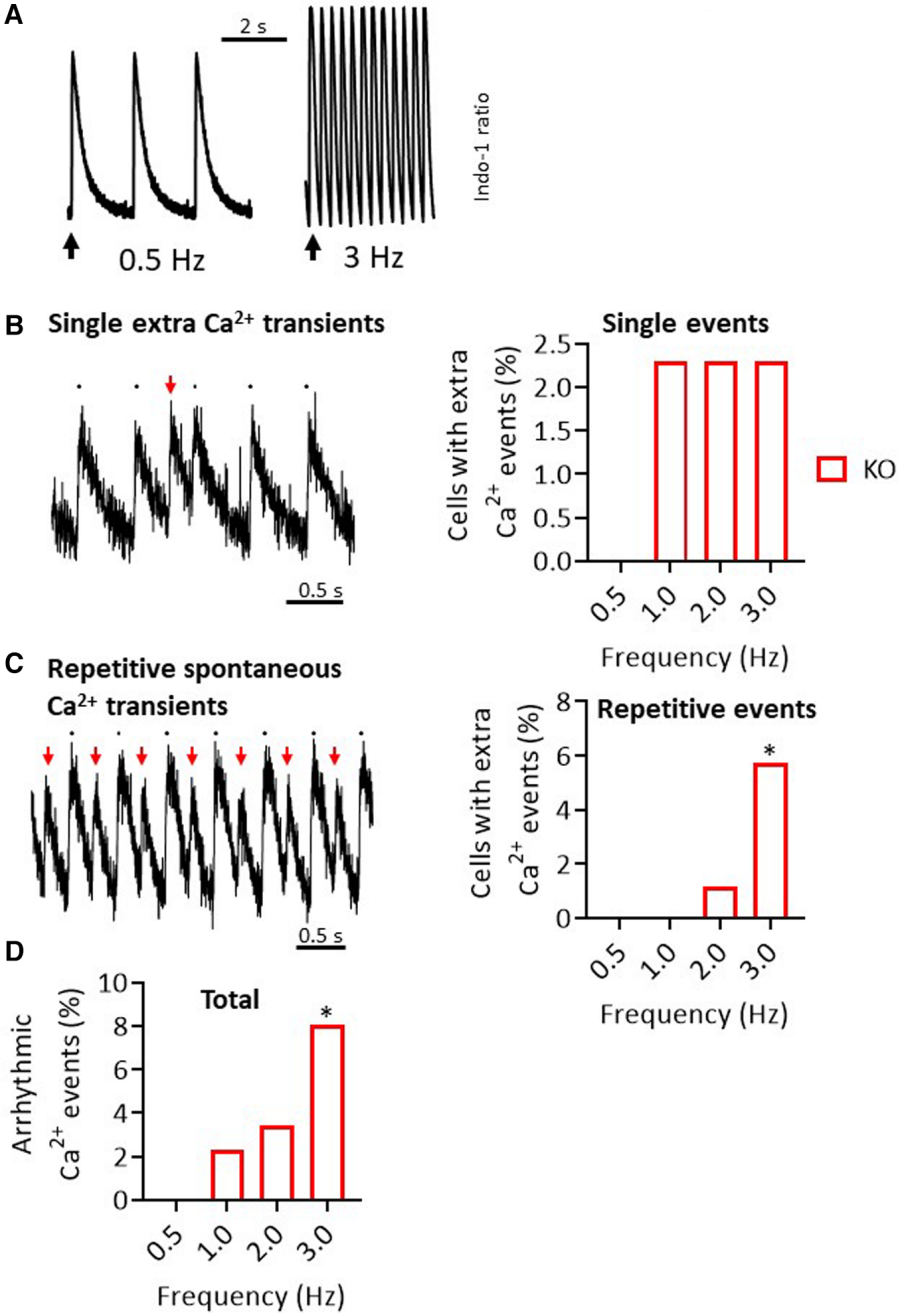
Increased rate of Ca^2+^ transient events in KO cardiomyocytes. (**A**) Representative representation of Ca^2+^ transients in the form of an Indo-1 ratio at different pacing frequencies in a WT cardiomyocyte. (**B**) In addition to the peaks at the stimulation times, isolated single extra-systolic Ca^2+^ events were recorded (left panel). These occurred with a certain frequency only in KO cells (right panel). (**C**) As a second pattern of Ca^2+^ events, repetitive spontaneous Ca^2+^ transients were observed (left panel). This phenomenon increased under higher stimulation frequencies, but only in KO (right panel) (*P < 0.05 vs. WT, Fisher's exact test). (**D**) Shown are the summed Ca^2+^ events (*P < 0.05 vs. WT, *n* = 49-63 cardiomyocytes/*N* = 5–6 mice, Fisher's exact test).

In search of the functional causes for the increased arrhythmic activity in KO, which was observed both under catecholamine administration (ECG) and under a pacing protocol (PES), we investigated intracellular Ca^2+^ handling by transient measurement in isolated cardiomyocytes. For this purpose, an established stimulation protocol (18 and Figure 6A) was chosen and Ca^2+^ transient peaks occurring apart from regular stimulation were counted. In WT cells, no additional Ca^2+^ events could be registered under any of the selected conditions. In contrast, KO cardiomyocytes showed single extra-systolic Ca^2+^ transients (Figure 6B) as well as regular arrhythmia in form of repetitive spontaneous Ca^2+^ transients under increasing frequencies up to 3 Hz (Figure 6C). In total, 8% of the measured KO cells showed different forms of arrhythmic events at cellular level (Figure 6D).

## Discussion

In the current study, we demonstrated that silencing of the *PPP2R5A gene* in a global knockout (KO) mouse model is associated with an increased propensity for the development of ventricular arrhythmias under catecholamergic stress in ECG measurements. The KO mice also showed increased ventricular arrhythmias in isolated Langendorff-perfused hearts in MAP measurements under programmed electrical stimulation (PES). Nevertheless, the arrhythmogenic phenotype was only slightly pronounced in both stress models. Moreover, the duration of MAP was unchanged between genotypes. Consistent with this, we also measured unchanged action potential duration and comparable potassium channel currents (*I*_K,total_) in cardiomyocytes from KO and WT. However, increased rates of arrhythmogenic Ca^2+^ events were seen at the single cell level in KO at increasing pacing frequencies.

A modified function of RyR2 is associated with an increased SR Ca^2+^ leak, which may contribute to an increased tendency to arrhythmias. Altered phosphorylation of the RyR2 was hypothesized as a possible explanation for dysfunction of the Ca^2+^ release channel. The basis for this assumption was the work of Marx and colleagues, who hypothesized a hyperadrenergic state as occurs in heart failure and during extreme stress (5). Since fatal ventricular arrhythmias occur particularly in the wake of heart failure, the causes and consequences of RyR2 dysfunction were of particular interest. The aforementioned catecholaminergic stress in heart failure leads to hyperphosphorylation of RyR2 serine residues 2030 and 2808, which results in dissociation of FKBP12 from RyR2 (5). This process results in leaky channels, i.e., the Ca^2+^ release channels tend to open even in the resting state. Adrenergic stress in turn reinforces this mechanism, resulting in an increased risk of arrhythmias. Later, other research groups were unable to detect such PKA-dependent hyperphosphorylation (35). In contrast, CaMKII-dependent hyperhosphorylation at serine residue 2814 of RyR2 was shown to be the cause of increased SR Ca^2+^ leak and the development of arrhythmias (36–39). Indeed, increased phosphorylation of RyR2 at the CaMKII site, but not at the PKA sites, was associated with increased Ca^2+^ spark frequency and arrhythmogenic oscillations of intracellular Ca^2+^ in a miR-1 overexpression model (40). Our B56α KO mice also show all the molecular and functional characteristics of a proarrhythmogenic substrate, namely increased phosphorylation of RyR2 at serine-2814 and increased Ca^2+^ spark frequency under adrenergic stress (18) as well as increased extra Ca^2+^ transient events with increase in pacing frequency (Figure 6). Both protocols, the acute administration of isoprenaline or the increase in stimulation frequency, ultimately lead to an increased activation of CaMKII (41). An increase in CaMKII activity, which was associated with a higher phosphorylation of RyR2 at serine-2814, was also shown in a mouse infarction model with ventricular arrhythmias (39). Interestingly, the cardiac phenotype was associated with reduced B56α protein expression. However, whether the additionally observed increase in phosphorylation of the LTCC under catecholaminergic stimulation in our model also contributes to the increased arrhythmogenicity due to increased Ca^2+^-induced Ca^2+^ release from the SR remains questionable, because the maximum current density of *I*_CaL_ was unchanged under this condition (18).

However, the overall degree of phosphorylation of RyR2 is not only determined by PKA or CaMKII activities, but also by dephosphorylation processes. Besides PP1, PP2A is responsible for the dephosphorylation of RyR2 at the CaMKII site, which plays an important role in increasing the Ca^2+^ leak from the SR. Paradoxically, PP2A increases the Ca^2+^ spark frequency in saponin-permeabilized rat cardiomyocytes after 1 min, but after 5 min the parameter falls below baseline (42). The fact that the administration of okadaic acid, a potent inhibitor of PP2A, has no effect on Ca^2+^ release suggests that the effect of PP2A on RyR2 must be mediated via anchor proteins (43). To date, it is known that PP2A is targeted to RyR2 either via B56α binding to ankyrin-B or binding of B56*δ* to the muscle-specific A-kinase-anchoring protein (mAKAP) (40, 44). The functional relevance of PP2A, regulatory subunit and anchor protein for the activity of RyR2 and thus for the development of proarrhythmic activity is demonstrated by an ankyrin-B loss-of-function mutant mouse model (17). This model shows an increased phosphorylation of serine-2814 under basal conditions and even more after administration of isoprenaline, increased Ca^2+^ waves, increased Ca^2+^ spark and DAD frequency and an increased predisposition to VTs. Of particular interest is that hyperphosphorylation of RyR2 is associated with increased dissociation of PP2A from the Ca^2+^ release channel and of B56α to ankyrin-B. Consistent with this, decreased expression of ankyrin-B resulted in a disorganized distribution of B56α in cardiomyocytes (45) and the promotion of increased cardiac arrhythmogenic activity (46) Similarly, in a canine heart failure model, increased phosphorylation levels of RyR2 at serine-2814 were associated with reduced association of PP2A-B56α at the Ca^2+^ release channel (16). In addition, Ca^2+^ wave frequencies and DAD rate were increased in the failing hearts. Thus, the decreased expression of B56α in human failing hearts (47) could contribute to the observed arrhythmic tendency following heart failure. Comparable results were also obtained by overexpression of miR-1 in rat ventricular cardiomyocytes, where the promotion of cardiac arrhythmogenesis was associated with a decrease in B56α protein expression (40). In a myocardial infarction model using left coronary ligation, the higher rate of spontaneous ventricular arrhythmias was also accompanied by reduced B56α expression (39). In contrast, increased PP2A activity resulted in reduced Ca^2+^ waves and sparks as well as a decrease in RyR2 phosphorylation (12). Thus, if reduced availability of catalytic PP2A at the Ca^2+^ release channel, either due to reduced expression of B56α *per se*, impaired expression/function of the anchor protein ankyrin-B or altered distribution/localization (in terms of dissociation from RyR2) after sympathetic stimulation (48), contributes to a CaMKII-dependent hyperphosphorylation of RyR2, it is not surprising that there is also an increased occurrence of VTs in our *PPP2R5A* KO model. The arrhythmic events are exclusively driven by uncontrolled and increased Ca^2+^ release from the SR, because both the duration of MAPs and the *I*_K,total_ are unchanged in KO hearts and single cells.

The line of evidence of reduced B56α and targeted PP2A activity, hyperphosphorylation of RyR2, extra Ca^2+^ transient events and the final triggering of VTs leads to approaches for targeted therapy of heart failure associated VTs or inherited arrhythmias that go beyond general antisympathetic therapy with β-blockers upstream of the myocardial cell. The latter demonstrated their efficacy in an ankyrin-B syndrome model in which metoprolol reduced the rate of VTs (17). The administration of propranolol reduced the phosphorylation of RyR2 in a diabetes-induced heart failure model (49). In addition, an increase in the expression of the RyR2 anchor protein FKBP12.6 was observed. Moreover, non-selective β-blockers also showed their effect in catecholaminergic polymorphic ventricular tachycardia (CPVT), a potentially life-threatening channelopathy with mutations of the CASQ2 and RyR2 genes (50). Successful treatment with β-blockers to minimize or terminate fatal arrhythmias has also been demonstrated for arrhythmogenic right ventricular cardiomyopathy (ARVC) or hypertrophic cardiomyopathy (HCM) (50). Within the cardiomyocytes, it may be possible to prevent the transfer of adrenergic/chronotropic triggers to intracellular signaling via CaMKII inhibition (51). The use of AC3I resulted in reduced phosphorylation of RyR2 at serine-2814 and a decrease in DAD events in a heterozygous ankyrin-B KO model. However, it remains questionable whether blanket activation of PP2A activity, e.g., through the use of fingolimod, is suitable for reducing or even preventing the tendency to arrhythmias, as the functions and regulation of the PP2A holoenzyme are too complex and diverse. Instead, active substances are needed that carefully and precisely direct PP2A to specific functions at the molecular level. So-called small-molecule activators of PP2A (SMAPs, e.g., DT-061; derived from phenothiazines), which are intended to stabilize the complex of PP2A and B56α in a target-specific manner, could contribute to this (52). The risks of such a therapy could lie in the occurrence of side effects due to the undirected differential expression of various PP2A subunits. Nevertheless, in contrast to the general adrenergic inhibition by β-blockers, the therapeutic benefit of using SMAPs could lie in a more local, molecule-specific inhibition of the higher SR Ca^2+^ release in ventricular arrhythmias by stabilizing the RyR2-PP2Ac-B56α complex. The use of selective phosphatase disrupting peptides, which lead to a reduction in arrhythmogenic Ca^2+^ events in end-stage heart failure cardiomyocytes, points in the same direction (53).

Despite the comprehensive methodological approach to clarifying the study's issues, there are obviously also some limitations. This concerns in particular the potential problems associated with the use and characterization of genetically modified mouse models. In general, it has now been demonstrated that such mouse models are effective tools for elucidating the functional and molecular mechanisms in both healthy and diseased myocardium. However, knocking out a gene in the whole organism does not necessarily lead to the expected cardiac phenotype. Thus, deletion of individual genes could lead to potential non-specific effects, such as buffering effects, counter-regulation of related genes or over-interpretation of certain signaling pathways. However, whether the increased protein expression of B56β and B56γ in *PPP2R5A* KO mice (18) contributes to the electrophysiological phenotype remains to be determined.

In conclusion, these results demonstrate for the first time that the development of a mild manifestation of ventricular tachyarrhythmias may result from a loss of PP2A-B56α expression in KO mice. This is due to proarrhythmogenic extra Ca^2+^ events in isolated cardiomyocytes. Our findings provide a direct rationale for establishing PP2A-B56α as a target for antiarrhythmic therapy. The testing of such agents, such as SMAPs, will be the goal of future studies.

## Data Availability

The raw data supporting the conclusions of this article will be made available by the authors, without undue reservation.

## References

[1] Janse MJ. Electrophysiological changes in heart failure and their relationship to arrhythmogenesis. Cardiovasc Res. (2004) 61(2):208–17. 10.1016/j.cardiores.2003.11.018 14736537

[2] Pogwizd SM Bers DM. Cellular basis of triggered arrhythmias in heart failure. Trends Cardiovasc Med. (2004) 14(2):61–6. 10.1016/j.tcm.2003.12.002 15030791

[3] Laurita KR Rosenbaum DS. Mechanisms and potential therapeutic targets for ventricular arrhythmias associated with impaired cardiac calcium cycling. J Mol Cell Cardiol. (2008) 44(1):31–43. 10.1016/j.yjmcc.2007.10.012 18061204 PMC2761085

[4] Bers DM. Cardiac excitation-contraction coupling. Nature. (2002) 415(6868):198–205. 10.1038/415198a 11805843

[5] Marx SO Reiken S Hisamatsu Y Jayaraman T Burkhoff D Rosemblit N PKA phosphorylation dissociates FKBP12.6 from the calcium release channel (ryanodine receptor): defective regulation in failing hearts. Cell. (2000) 101(4):365–76. 10.1016/s0092-8674(00)80847-8 10830164

[6] Kockskämper J Pieske B. Phosphorylation of the cardiac ryanodine receptor by Ca^2+^/calmodulin-dependent protein kinase II: the dominating twin of protein kinase a? Circ Res. (2006) 99(4):333–5. 10.1161/01.RES.0000239406.66844.7d 16917097

[7] Lehnart SE Wehrens XH Kushnir A Marks AR. Cardiac ryanodine receptor function and regulation in heart disease. Ann N Y Acad Sci. (2004) 1015:144–59. 10.1196/annals.1302.012 15201156

[8] George CH Higgs GV Lai FA. Ryanodine receptor mutations associated with stress-induced ventricular tachycardia mediate increased calcium release in stimulated cardiomyocytes. Circ Res. (2003) 93(6):531–40. 10.1161/01.RES.0000091335.07574.86 12919952

[9] Klapproth E Kämmerer S El-Armouche A. Function and regulation of phosphatase 1 in healthy and diseased heart. Cell Signal. (2022) 90:110203. 10.1016/j.cellsig.2021.110203 34822978

[10] Heijman J Dewenter M El-Armouche A Dobrev D. Function and regulation of serine/threonine phosphatases in the healthy and diseased heart. J Mol Cell Cardiol. (2013) 64:90–8. 10.1016/j.yjmcc.2013.09.006 24051368

[11] Potenza DM Janicek R Fernandez-Tenorio M Niggli E. Activation of endogenous protein phosphatase 1 enhances the calcium sensitivity of the ryanodine receptor type 2 in murine ventricular cardiomyocytes. J Physiol. (2020) 598(6):1131–50. 10.1113/JP278951 31943206

[12] Little SC Curran J Makara MA Kline CF Ho HT Xu Z Protein phosphatase 2A regulatory subunit B56α limits phosphatase activity in the heart. Sci Signal. (2015) 8(386):ra72. 10.1126/scisignal.aaa5876 26198358 PMC4680974

[13] DeGrande ST Little SC Nixon DJ Wright P Snyder J Dun W Molecular mechanisms underlying cardiac protein phosphatase 2A regulation in heart. J Biol Chem. (2013) 288(2):1032–46. 10.1074/jbc.M112.426957 23204520 PMC3542989

[14] Reynhout S Janssens V. Physiologic functions of PP2A: lessons from genetically modified mice. Biochim Biophys Acta. (2019) 1866(1):31–50. 10.1016/j.bbamcr.2018.07.010 30030003

[15] Hall DD Feekes JA Arachchige Don AS Shi M Hamid J Chen L Binding of protein phosphatase 2A to the L-type calcium channel Cav1.2 next to Ser1928, its main PKA site, is critical for Ser1928 dephosphorylation. Biochemistry. (2006) 45(10):3448–59. 10.1021/bi051593z 16519540

[16] Belevych AE Sansom SE Terentyeva R Ho HT Nishijima Y Martin MM MicroRNA-1 and −133 increase arrhythmogenesis in heart failure by dissociating phosphatase activity from RyR2 complex. PLoS One. (2011) 6(12):e28324. 10.1371/journal.pone.0028324 22163007 PMC3232211

[17] Zhu W Wang C Hu J Wan R Yu J Xie J Ankyrin-B Q1283H variant linked to arrhythmias via loss of local protein phosphatase 2A activity causes ryanodine receptor hyperphosphorylation. Circulation. (2018) 138(23):2682–97. 10.1161/CIRCULATIONAHA.118.034541 30571258 PMC6276866

[18] Glaser D Heinick A Herting JR Massing F Müller FU Pauls P Impaired myocellular Ca^2+^ cycling in protein phosphatase PP2A-B56α KO mice is normalized by β-adrenergic stimulation. J Biol Chem. (2022) 298(9):102362. 10.1016/j.jbc.2022.102362 35963431 PMC9478386

[19] Schulte JS Fehrmann E Tekook MA Kranick D Fels B Li N Cardiac expression of the CREM repressor isoform CREM-IbΔC-X in mice leads to arrhythmogenic alterations in ventricular cardiomyocytes. Basic Res Cardiol. (2016) 111(2):15. 10.1007/s00395-016-0532-y 26818679 PMC4729809

[20] Barold SS. Willem Einthoven and the birth of clinical electrocardiography a hundred years ago. Card Electrophysiol Rev. (2003) 7(1):99–104. 10.1023/a:1023667812925 12766530

[21] Boukens BJ Rivaud MR Rentschler S Coronel R. Misinterpretation of the mouse ECG: “musing the waves of mus musculus”. J Physiol. (2014) 592(21):4613–26. 10.1113/jphysiol.2014.279380 25260630 PMC4253466

[22] Calvet C Seebeck P. What to consider for ECG in mice-with special emphasis on telemetry. Mamm Genome. (2023) 34(2):166–79. 10.1007/s00335-023-09977-0 36749381 PMC10290603

[23] Oestereicher MA Wotton JM Ayabe S Bou About G Cheng TK Choi JH Comprehensive ECG reference intervals in C57BL/6N substrains provide a generalizable guide for cardiac electrophysiology studies in mice. Mamm Genome. (2023) 34(2):180–99. 10.1007/s00335-023-09995-y 37294348 PMC10290602

[24] Salama G London B. Mouse models of long QT syndrome. J Physiol. (2007) 578(Pt 1):43–53. 10.1113/jphysiol.2006.118745 17038432 PMC2075110

[25] Speerschneider T Thomsen MB. Physiology and analysis of the electrocardiographic T-wave in mice. Acta Physiol. (2013) 209(4):262–71. 10.1111/apha.12172 24119104

[26] Herting JR König JH Hadova K Heinick A Müller FU Pauls P Hypercontractile cardiac phenotype in mice overexpressing the regulatory subunit PR72 of protein phosphatase 2A. Front Cardiovasc Med. (2023) 10:1239555. 10.3389/fcvm.2023.1239555 37868783 PMC10590119

[27] Knollmann BC Katchman AN Franz MR. Monophasic action potential recordings from intact mouse heart: validation, regional heterogeneity, and relation to refractoriness. J Cardiovasc Electrophysiol. (2001) 12(11):1286–94. 10.1046/j.1540-8167.2001.01286.x 11761418

[28] Stokoe KS Balasubramaniam R Goddard CA Colledge WH Grace AA Huang CL. Effects of flecainide and quinidine on arrhythmogenic properties of *Scn5a*+/− murine hearts modelling the Brugada syndrome. J Physiol. (2007) 581(Pt 1):255–75. 10.1113/jphysiol.2007.128785 17303635 PMC2075209

[29] Balasubramaniam R Grace AA Saumarez RC Vandenberg JI Huang CL. Electrogram prolongation and nifedipine-suppressible ventricular arrhythmias in mice following targeted disruption of KCNE1. J Physiol. (2003) 552(Pt 2):535–46. 10.1113/jphysiol.2003.048249 14561835 PMC2343378

[30] Kučerová D Baba HA Bokník P Fabritz L Heinick A Mát’uš M Modulation of SR Ca^2+^ release by the triadin-to-calsequestrin ratio in ventricular myocytes. Am J Physiol Heart Circ Physiol. (2012) 302(10):H2008–17. 10.1152/ajpheart.00457.2011 22427521

[31] Roden DM. Keep the QT interval: it is a reliable predictor of ventricular arrhythmias. Heart Rhythm. (2008) 5(8):1213–5. 10.1016/j.hrthm.2008.05.008 18675237 PMC3212752

[32] Fabritz L Kirchhof P Franz MR Eckardt L Mönnig G Milberg P Prolonged action potential durations, increased dispersion of repolarization, and polymorphic ventricular tachycardia in a mouse model of proarrhythmia. Basic Res Cardiol. (2003) 98(1):25–32. 10.1007/s00395-003-0386-y 12494266

[33] Killeen MJ Thomas G Gurung IS Goddard CA Fraser JA Mahaut-Smith MP Arrhythmogenic mechanisms in the isolated perfused hypokalaemic murine heart. Acta Physiol. (2007) 189(1):33–46. 10.1111/j.1748-1716.2006.01643.x PMC1859975 17280555

[34] Decher N Wemhöner K Rinné S Netter MF Zuzarte M Aller MI Knock-out of the potassium channel TASK-1 leads to a prolonged QT interval and a disturbed QRS complex. Cell Physiol Biochem. (2011) 28(1):77–86. 10.1159/000331715 21865850

[35] Xiao B Jiang MT Zhao M Yang D Sutherland C Lai FA Characterization of a novel PKA phosphorylation site, serine-2030, reveals no PKA hyperphosphorylation of the cardiac ryanodine receptor in canine heart failure. Circ Res. (2005) 96(8):847–55. 10.1161/01.RES.0000163276.26083.e8 15790957

[36] Wehrens XH Lehnart SE Reiken SR Marks AR. Ca^2+^/calmodulin-dependent protein kinase II phosphorylation regulates the cardiac ryanodine receptor. Circ Res. (2004) 94(6):e61–70. 10.1161/01.RES.0000125626.33738.E2 15016728

[37] Ai X Curran JW Shannon TR Bers DM Pogwizd SM. Ca^2+^/calmodulin-dependent protein kinase modulates cardiac ryanodine receptor phosphorylation and sarcoplasmic reticulum Ca2 + leak in heart failure. Circ Res. (2005) 97(12):1314–22. 10.1161/01.RES.0000194329.41863.89 16269653

[38] Curran J Brown KH Santiago DJ Pogwizd S Bers DM Shannon TR. Spontaneous Ca waves in ventricular myocytes from failing hearts depend on Ca(2+)-calmodulin-dependent protein kinase II. J Mol Cell Cardiol. (2010) 49(1):25–32. 10.1016/j.yjmcc.2010.03.013 20353795 PMC2883657

[39] Qin R Murakoshi N Xu D Tajiri K Feng D Stujanna EN Exercise training reduces ventricular arrhythmias through restoring calcium handling and sympathetic tone in myocardial infarction mice. Physiol Rep. (2019) 7(4):e13972. 10.14814/phy2.13972 30806037 PMC6389758

[40] Terentyev D Belevych AE Terentyeva R Martin MM Malana GE Kuhn DE miR-1 overexpression enhances Ca(2+) release and promotes cardiac arrhythmogenesis by targeting PP2A regulatory subunit B56alpha and causing CaMKII-dependent hyperphosphorylation of RyR2. Circ Res. (2009) 104(4):514–21. 10.1161/CIRCRESAHA.108.181651 19131648 PMC4394868

[41] Valverde CA Mundiña-Weilenmann C Said M Ferrero P Vittone L Salas M Frequency-dependent acceleration of relaxation in mammalian heart: a property not relying on phospholamban and SERCA2a phosphorylation. J Physiol. (2005) 562(Pt 3):801–13. 10.1113/jphysiol.2004.075432 15528241 PMC1665530

[42] Terentyev D Viatchenko-Karpinski S Gyorke I Terentyeva R Gyorke S. Protein phosphatases decrease sarcoplasmic reticulum calcium content by stimulating calcium release in cardiac myocytes. J Physiol. (2003) 552(Pt 1):109–18. 10.1113/jphysiol.2003.046367 12897175 PMC2343319

[43] Lubbers ER Mohler PJ. Roles and regulation of protein phosphatase 2A (PP2A) in the heart. J Mol Cell Cardiol. (2016) 101:127–33. 10.1016/j.yjmcc.2016.11.003 27832939 PMC5939568

[44] Dodge-Kafka KL Bauman A Mayer N Henson E Heredia L Ahn J cAMP-stimulated protein phosphatase 2A activity associated with muscle A kinase-anchoring protein (mAKAP) signaling complexes inhibits the phosphorylation and activity of the cAMP-specific phosphodiesterase PDE4D3. J Biol Chem. (2010) 285(15):11078–86. 10.1074/jbc.M109.034868 20106966 PMC2856983

[45] Bhasin N Cunha SR Mudannayake M Gigena MS Rogers TB Mohler PJ. Molecular basis for PP2A regulatory subunit B56alpha targeting in cardiomyocytes. Am J Physiol Heart Circ Physiol. (2007) 293(1):H109–19. 10.1152/ajpheart.00059.2007 17416611

[46] Camors E Mohler PJ Bers DM Despa S. Ankyrin-B reduction enhances Ca spark-mediated SR Ca release promoting cardiac myocyte arrhythmic activity. J Mol Cell Cardiol. (2012) 52(6):1240–8. 10.1016/j.yjmcc.2012.02.010 22406428 PMC3348355

[47] Wijnker PJ Boknik P Gergs U Müller FU Neumann J dos Remedios C Protein phosphatase 2A affects myofilament contractility in non-failing but not in failing human myocardium. J Muscle Res Cell Motil. (2011) 32(3):221–33. 10.1007/s10974-011-9261-x 21959857 PMC3205269

[48] Yin X Cuello F Mayr U Hao Z Hornshaw M Ehler E Proteomics analysis of the cardiac myofilament subproteome reveals dynamic alterations in phosphatase subunit distribution. Mol Cell Proteomics. (2010) 9(3):497–509. 10.1074/mcp.M900275-MCP200 20037178 PMC2849712

[49] Tuncay E Zeydanli EN Turan B. Cardioprotective effect of propranolol on diabetes-induced altered intracellular Ca^2+^ signaling in rat. J Bioenerg Biomembr. (2011) 43(6):747–56. 10.1007/s10863-011-9400-5 22127436

[50] Mariani MV Pierucci N Fanisio F Laviola D Silvetti G Piro A Inherited arrhythmias in the pediatric population: an updated overview. Medicina. (2024) 60(1):94. 10.3390/medicina60010094 38256355 PMC10819657

[51] DeGrande S Nixon D Koval O Curran JW Wright P Wang Q CaMKII inhibition rescues proarrhythmic phenotypes in the model of human ankyrin-B syndrome. Heart Rhythm. (2012) 9(12):2034–41. 10.1016/j.hrthm.2012.08.026 23059182 PMC3630478

[52] Leonard D Huang W Izadmehr S O’Connor CM Wiredja DD Wang Z Selective PP2A enhancement through biased heterotrimer stabilization. Cell. (2020) 181(3):688–701.e16. 10.1016/j.cell.2020.03.038 32315618 PMC7243596

[53] Fischer TH Eiringhaus J Dybkova N Saadatmand A Pabel S Weber S Activation of protein phosphatase 1 by a selective phosphatase disrupting peptide reduces sarcoplasmic reticulum Ca^2+^ leak in human heart failure. Eur J Heart Fail. (2018) 20(12):1673–85. 10.1002/ejhf.1297 30191648

